# Does plain radiography still have a role in cases of fish bone ingestion in emergency rooms? A retrospective analysis

**DOI:** 10.1007/s10140-020-01891-1

**Published:** 2021-01-27

**Authors:** Tzu-Chi Wu, Pin-Wen Huang, Chun-Bin Tung

**Affiliations:** 1 Department of Emergency Medicine, Show Chwan Memorial Hospital, Changhua, Taiwan; 2 Graduate Institute of Technology Management, National Chung Hsing University, Taichung, Taiwan

**Keywords:** Fish bone, Foreign body, Lateral neck radiography, Radiography in emergency care

## Abstract

**Background:**

Fish bones are the most common aerodigestive foreign bodies found in adults. Most cases of fish bone impaction improve after primary management by emergency physicians with a mirror laryngoscopy using a tongue depressor, before otolaryngologists perform a fiberoptic nasendoscopy. A computed tomography scan usually follows to determine the next step. Studies have recently been concerned about overdoses of radiation from computed tomography. However, clear algorithms remain unavailable for fish bone ingestion management to date.

**Methods:**

A retrospective review was conducted on 180 patients who visited the emergency department with complaints of fish bone impaction between January 2017 and January 2019.

**Results:**

A total of 81.6% of patients with fish bone impaction got symptomatic relief after primary management by emergency physicians and otolaryngologists. Out of 180 patients, 33 (18.3%) needed an endoscopic procedure due to persistent symptoms. Only one (0.56%) required an operation due to perforation. In the group failing primary management, the plain radiography of eight patients showed a positive finding and an esophagogastroscopy was done to remove the fish bones.

**Conclusion:**

Lateral neck radiography is still beneficial to patients with fish bone ingestion failure from primary management. Positive lateral soft tissue radiography in cases with persistent symptoms post primary management may directly suggest esophagogastroscopy without confirmation from a computed tomography, unless complications are suspected. For patients aged below 40, following up on their conditions after post management radiography shows negative results may increase their safety.

## Introduction

Foreign body impaction is a common reason for emergency room visits. A direct visualization of the fishbone is the best diagnostic method and can also resolve patient anxiety. On one hand, plain lateral neck radiography makes locating fish bones difficult, because not all of them are radio opaque. On the other hand, computed tomography is effective at identifying impacted esophageal fish bones with high sensitivity. We cannot exclude fish bone impaction due to minimal false-negative rates, especially when symptoms persist. Therefore, endoscopic intervention plays a definite role in patients with persistent symptoms or whose computed tomography scan is positive. There have so far not been any clear algorithms available for fish bone impaction diagnosis and management. Our study fills this gap by reviewing cases of fish bone impaction and proposing a management protocol to ensure an optimum outcome for patients.

## Methods

**Fig. 1:**
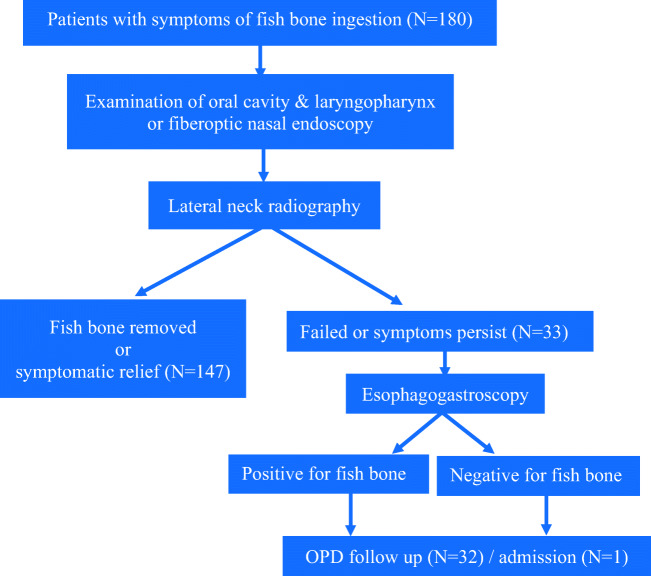
Fish bone foreign body management algorithm

A retrospective chart review was conducted on 180 patients who complained of fish bone impaction in Taiwan’s Show Chwan Memorial Hospital between January 2017 and 2019. Patients with suspected fish bones in their throats underwent a mirror laryngoscopy with the use of a tongue depressor, followed by a fiberoptic nasendoscopy performed by emergency physicians, then otolaryngologists. A lateral neck radiography was also done. After primary management, patients may be discharged if the foreign body is removed, or if there is symptomatic relief regardless of removal of a foreign body. Failing primary management means symptoms persist post both emergency physician and otolaryngologist management. If symptoms persisted, an esophagogastroscopy intervention was arranged (Fig. 1). We used the Mann-Whitney *U* test and the Fisher exact test. All statistical analyses were done using the IBM SPSS ver. 20.0. The statistical significance threshold was set at < 0.05 (two-sided test).

## Results

Between January 2017 and January 2019, 147 (81.6%) out of 180 total cases of fish bone foreign body ingestion gained symptomatic relief after the oral cavity or laryngopharynx was examined by emergency physicians or otolaryngologists. Thirty-three (18.3%) patients needed an endoscopic procedure due to persistent symptoms. Only one (0.56%) required an operation due to perforation as the foreign body had been in the patient’s system for over 3 days. In this case, a computed tomography was performed instead.

**Fig. 2:**
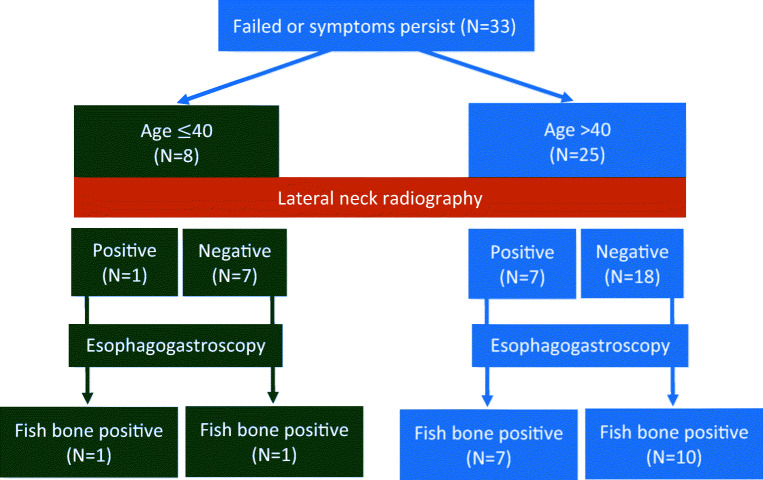
Failure after primary management

Among the 33 (18.3%) patients whose symptoms persisted post primary management were 14 men, 19 women, 8 patients below 40 years old, and 25 patients above 40. The mean age was 54.1 years (SD, ± 16.9) and body mass index was 24.1 (SD, ± 3.94) (Table 1). Out of all the patients (*N* = 33), 8 (24.2%) showed a positive finding from plain radiography, and 100% of patients had a fish bone removed via esophagogastroscopy (Fig. 2). The age of the group that needed an esophagogastroscopy procedure was higher than that of the group which had fish bones removed post primary management (54.18/42.25, *P* = 0.012). Additionally, positive findings of lateral neck radiography have a higher percentage (24.2%) in the esophagogastroscopy procedure group (*P* = 0.035) (Table 1).

**Table 1:** Attributes of patients treated during primary management vs patients needing esophagogastroscopy

	Removed by mirror laryngoscopy/fiberoptic nasendoscopy	Esophagogastroscopy	*p* value
Number	147 (81.6%)	33(18.3%)	
Age	42.25 ± 23.93	54.18 ± 16.91	0.012
Age ≤ 40	65 (44.3%)	8 (24.2%)	
Age > 40	82 (55.7%)	25 (75.8%)	0.035
Plain radiography positive	13 (8.8%)	8 (24.2%)	0.035
BMI	23.04 ± 5.29	24.10 ± 3.94	0.406

Foreign bodies were successfully removed by esophagogastroscopy in 19 cases, which is 10.5% of the total number of patients. In this sample (*N* = 19), there were 9 men and 10 women, 2 patients were younger than 40 years of age, and 17 were older. Their mean age was 57.4 (SD, ± 13.5) and body mass index (BMI) was 24.3 (SD, ± 4.04). The fish bone was located in the vallecula or pyriform sinus of 5 patients, in the upper third of the esophagus of 13 (68.4%) patients, and in the middle third of the esophagus in 1 (5.2%) patient. The average length of the fish bone was 2.05 cm. The timing of endoscopic intervention was 64.1 min on average, after the patient arrived at the emergency department. Moreover, fish bones were more commonly found by esophagogastroscopy in cases where patients were above 40 years old (*P* = 0.047) and in patients with positive findings on lateral neck radiography (*P* = 0.01) (Table 2). The difference was significant.

**Table 2:** Attributes of patients who underwent esophagogastroscopy and fish bone location

Esophagogastroscopy	Positive with fish bone (*N* = 19)	Negative with fish bone (*N* = 14)	*p*-value
Sex	Male 9 (47.4%)	Male 5 (35.7%)	
	Female 10 (52.6%)	Female 9 (64.3%)	
Age	57.42 ± 13.56	49.79 ± 20.33	0.209
Age ≤ 40	2(10.6%)	6(42.9%)	0.047
Age > 40	17(89.4%)	8(57.1%)	
BMI	24.28 ± 4.04	23.84 ± 4.17	0.814
Plain radiography			
positive	8 (42.1%)	0 (0.0%)	0.010
negative	11(57.9%)	14(100%)	
Fish bone location	Vallecula / Pyriform sinus (*N* = 5) Upper third of the esophagus (*N* = 13) Middle third of the esophagus(*N* = 1)		

## Discussion

Up to 80% of patients can gain symptomatic relief after primary management by emergency physicians and the otolaryngologists. The most common impaction site of a fish bone is the oropharynx and oral cavity, followed by the esophagus. Within the esophagus, the upper third of the esophagus is the most common location. This may be due to the first physiological narrowing caused by the juncture with the pharynx. The risk of esophageal impaction needing endoscopic intervention increases with age. In our analysis, the risk of needing endoscopic intervention increases by 1.039 times for every 1 year of age, *P* = 0.024; the difference was significant. The BMI made no difference between the two groups needing further management or not.

Oropharynx/hypopharynx are more common locations, but plain radiography is less reliable in this area due to the high soft tissue and bone density over the suprahyoid area. Overall, the positive rate of lateral neck radiography was 11.6%. In a retrospective study of 698 cases with fish bones in the oropharynx [1], only 23 (10%) cases were reported by the radiologist as being positive for the presence of a fish bone. This result suggested that lateral soft tissue radiography is not beneficial in cases with a suspected fish bone when fiberoptic nasendoscope is available.

In most situations, a computed tomography scan is arranged when there is a lack of findings during the fiberoptic nasendoscopy if symptoms persisted due to high sensitivity and specificity. Thus, a high percentage of those who have persistent symptoms require further intervention. According to Kim et al. [2], 66 (75%) out of 88 patients with a fish bone in their esophagus underwent non-contrast-computed tomography because the foreign body was not removed through a laryngoscope. This suggests that up to 75% of patients need esophagogastroscopy if symptoms persist post laryngoscopic intervention. In our study, 19 (57.6%) of the 33 cases had the fish bone removed by esophagogastroscopy, excluding those with only esophageal abrasions, bleeding, or if the fish bone slipped out to the stomach.

Studies have recently discussed that many patients, especially younger ones, undergo unnecessary imaging. In a study with 286 total cases of fish bone foreign body ingestion [2], the positive rate was higher in 88 patients aged over 40 years with non-contrast-computed tomography; younger patients below 40 are far more likely to be impacted in the oropharynx. According to Klein [3], patients below 40 years old examined within 24 h from ingestion show little diagnostic value from imaging due to the low probability of esophageal fish bones. Many young patients undergo unnecessary computed tomography studies. Therefore, some have suggested that patients younger than 40, and visiting the emergency department less than 24 h after the fish bone ingestion, can be followed up after primary management without computed tomography.

Plain radiography has a much lower radiation dose than computed tomography scans. Radiation exposure is also an important concern, especially in young patients. According to most studies, it is not beneficial before primary management. Thus, it may have a role before the computed tomography scan or discharge in some conditions.

**Fig. 3:**
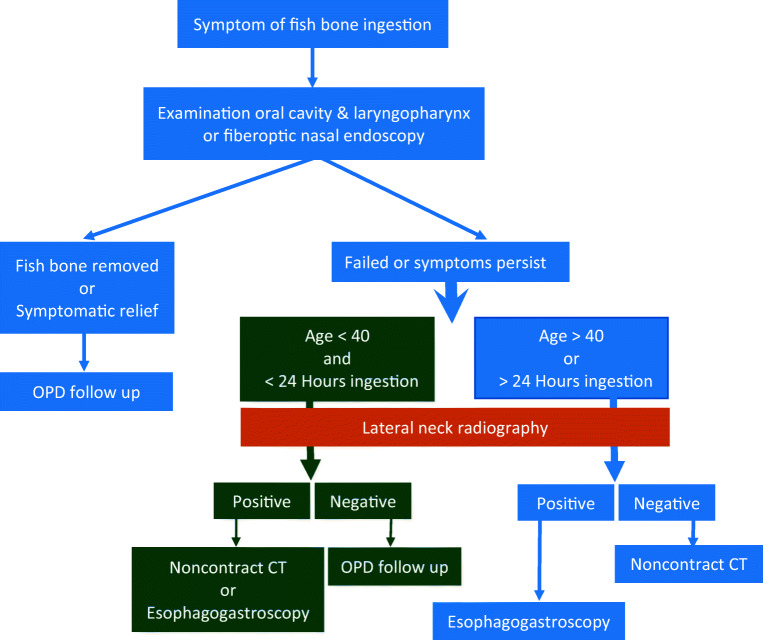
New management algorithm of fish bone foreign body

In the group that failed the post primary management, eight patients showed a positive finding in their plain radiography, and all of them underwent esophagogastroscopy to remove the fish bone (Fig. 2). This may suggest that if the lateral soft tissue radiography is positive post primary management, an esophagogastroscopy can be done without confirming the results by computed tomography. Moreover, up to 89% of cases in the group younger than 40 years old (*N* = 73) were resolved post primary management, while 8 (11%) need further management. Only two showed positive findings of fish bones by esophagogastroscopy. This suggests that it is safe to discharge patients with fish bone impaction according to the management algorithm by Klein [3]. However, one in two patients younger than 40 years showed positive findings of fish bones. If we check the plain radiography before discharge, one may directly arrange esophagogastroscopy without observation or a computed tomography scan if symptoms persist. Further, a computed tomography scan is unnecessary for the group aged above 40 whose plain radiography came out positive for the presence of fish bones, unless complications, such as abscess formation or perforation, are suspected (Fig. 3).

The timing of endoscopic intervention is associated with risks of aspiration and perforation. According to the European Society of Gastrointestinal Endoscopy guidelines, sharp-pointed objects such as fish bone foreign bodies are considered an emergency condition and should preferably be removed within 6 h at most. Esophageal foreign bodies without complete obstruction are considered an urgent condition that requires therapeutic esophagogastroduodenoscopy to be performed within 24 h [4]. Therefore, in situations where a computed tomography scan is not available within 24 h, lateral soft tissue radiography may improve the safety of patients and clear the pathway.

This study has several limitations. Primarily, the size of the study was small when compared with other studies. Second, it was a retrospective study which carries its own bias and weaknesses.

Up to 80% of the patients get symptomatic relief after management by emergency department physicians and then otolaryngologists. If the examination is unremarkable and symptoms persist, lateral neck radiography still plays a role. Plain radiography, showing positive in cases where symptoms persist post primary management, may be directly followed by an esophagogastroscopy without confirming a computed tomography, unless complications are suspected. For patients younger than 40 years old, outpatient department follow-up post negative of neck radiography may improve the safety of patients.
