# Correction to: Does plain radiography still have a role in cases of fish bone ingestion in emergency rooms? A retrospective analysis

**DOI:** 10.1007/s10140-021-01913-6

**Published:** 2021-02-11

**Authors:** Tzu-Chi Wu, Pin-Wen Huang, Chun-Bin Tung

**Affiliations:** 1 Department of Emergency Medicine, Show Chwan Memorial Hospital, Changhua, Taiwan; 2 Graduate Institute of Technology Management, National Chung Hsing University, Taichung, Taiwan

**Correction to: Emergency Radiology (2021).**

10.1007/s10140-020-01891-1

In the original version of this paper contains an error in Methods section. BLINDED text should be replaced with “Show Chwan Memorial Hospital”. The Original article has been corrected.

